# Improved generative adversarial networks model for movie dance generation

**DOI:** 10.1371/journal.pone.0323304

**Published:** 2025-05-23

**Authors:** Zhiqun Lin, Kexin Feng

**Affiliations:** Music College of Capital Normal University, Beijing, China; Helwan University Faculty of Art Education, EGYPT

## Abstract

To address the challenges of innovation and efficiency in film choreography, this study proposes a dance generation model based on the generative adversarial networks. The model is trained using the AIST++ dance motion dataset, incorporating data from multiple dance styles to ensure that the generated dance sequences accurately mimic various stylistic and technical characteristics. The model integrates a music synchronization mechanism and dance structure constraints. These features ensure that the generated dance aligns seamlessly with the background music in terms of rhythm and emotional expression. Additionally, they help maintain a coherent dance structure. Experimental results demonstrate that the proposed model achieves a peak signal-to-noise ratio of 28.5 dB in dance video generation, representing an improvement of 4.2 dB over traditional methods. The structural similarity index reaches 0.83, surpassing the 0.79 achieved by conventional approaches. In a blind evaluation, 85% of professional dancers found the generated dance sequences highly consistent with the original dance styles, marking a 13% improvement over traditional methods. These findings indicate that the model effectively captures the details and fluidity of complex dance movements, providing an innovative and efficient solution for film dance generation.

## 1. Introduction

In the realm of filmmaking, dance sequences serve to convey characters’ inner realms, propel narratives, or evoke specific atmospheres [1–3]. Dance in film is not merely a reflection of character or an expression of plot; it is a unique art form that enriches the visual experience of a film and enhances emotional resonance. Through meticulously choreographed dance movements, films can more effectively convey the emotions and conflicts within characters, allowing viewers to experience a deeper sense of connection. Furthermore, film dance serves not only as a form of entertainment but also as a vehicle for cultural heritage and artistic innovation. Chinese folk dance, in particular, emphasizes traditional cultural expression and regional characteristics. Although differences in form and purpose exist between the two, the interaction and fusion between film dance and traditional dance have infused modern dance with renewed vitality.

Traditional choreography methods typically rely on established principles and experience in dance composition, structuring movements through direct design, pattern layering, and arrangement. These methods emphasize the precision and procedural nature of dancers’ movements without necessarily incorporating advanced computational techniques or machine learning. In traditional choreography, creation is often driven by a choreographer’s deep understanding of dance steps and intuitive arrangement of body language. For instance, in ballet, contemporary dance, or folk dance choreography, choreographers sequentially add dance steps, technical movements, and emotional expressions to construct dance sequences that align with specific stylistic or narrative requirements. The core characteristics of these methods lie in the coordination between the dancer’s body and technical movements, as well as the intuitive and manually crafted choreography. From a technical perspective, traditional choreography methods demand high precision and technical proficiency from dancers, relying heavily on the choreographer’s in-depth understanding of bodily movement and creative expression. Therefore, technologies for generating dance in film play a significant role in advancing modern dance, fostering artistic innovation, and enhancing creative expression.

However, amidst the rapid evolution of the film industry and heightened expectations for visual effects, traditional methods of dance choreography confront unprecedented challenges in balancing creativity with production efficiency [4,5]. These methods traditionally rely on choreographers’ intuition and expertise, necessitating prolonged collaboration and experimentation among dancers and directors to achieve desired performances [6,7]. While fostering highly personalized and perceptibly human artistic expressions, this approach encounters significant constraints in terms of time management and cost containment [8–10]. Furthermore, ensuring consistency and replicability in each performance proves challenging, especially within the context of tightly synchronized film shoots [11]. Moreover, traditional dance choreography struggles to faithfully replicate intricate details and explore innovative dance styles, particularly in handling intricate dance movements and diverse expressive demands [12,13].

Dance generation in films presents a unique set of challenges compared to other dance styles, such as fitness dance, hip-hop, and ballet. First, dance in films is not merely a display of movements; it serves as an artistic expression that integrates with the storyline, character emotions, and cinematic atmosphere. In traditional dance generation, the primary focus is often on the execution of movements. However, in film applications, dance sequences must be closely aligned with the narrative structure to achieve both visual and emotional coherence. Particularly, at different stages of story progression and character emotional shifts, dance movements must adapt to psychological changes and convey the emotional dynamics of the plot. For instance, when a film depicts emotional conflict in a character, the dance must reflect these emotional layers through variations in rhythm, tension in postures, and the subtleties of emotional expression. This requirement makes dance generation more than just imitating movements—it necessitates a precise understanding of the film’s storyline, character personalities, and emotional fluctuations.

Moreover, emotional resonance and long-term continuity are two critical factors in film dance generation. Unlike other dance forms, film dance must maintain consistency in movement and emotion across multiple shots. Since dance sequences in films often span several scenes and camera angles, the generated dance must ensure smooth transitions and seamless continuity while aligning with the film’s overall emotional tone. For example, in a dance sequence that represents a protagonist’s emotional transformation, the changes in movement are not just physical but must also convey the character’s inner shift through rhythm, intensity, and form. Additionally, this transformation must remain consistent with the film’s narrative progression. The need for sustained emotional expression and synchronized storytelling makes dance generation for films significantly more complex than other dance styles.

In response to these challenges, artificial intelligence (AI) technologies, notably generative adversarial networks (GANs), have been introduced into dance creation as a transformative solution [14–16]. GANs operate as a dual-component machine learning framework comprising a generator and a discriminator that engage in mutual enhancement during the learning process [17,18]. Demonstrating substantial promise in fields such as image and video generation, as well as music composition, GANs facilitate the creation of both novel and authentically inspired artistic works [19].

Nevertheless, the utilization of GANs in the realm of film dance generation remains relatively limited, particularly in terms of research dedicated to upholding professionalism and stylistic refinement in dance [20]. Existing methodologies primarily focus on generating rudimentary movements rather than intricate, artistically sophisticated professional dances. Furthermore, ensuring precise synchronization of generated dance sequences with the film’s music and narrative remains a technical challenge that has yet to be fully resolved [21]. Existing models consider basic movement patterns for generating dance sequences. However, they do not adequately capture important technical details, such as body control, balance, and rhythmic expression, which are essential for achieving high-quality and aesthetically pleasing dance performances.

This study proposes an enhanced GANs model designed to generate professional-grade dance sequences specifically tailored for film production, referred to as “Movie Dance Generation” hereafter. This term encompasses the generation of stylized dance movements that align with the thematic and artistic requirements of cinematic narratives. By leveraging GANs, the proposed model not only meets the technical demands of professional choreography but also ensures synchronization with musical elements, offering a cohesive and expressive audiovisual experience for cinematic applications. The novel GANs model is developed utilizing publicly available datasets featuring diverse dance movements performed by professional dancers. The primary innovations and contributions of this study are as follows: (1) Design of a Specialized Model for Film Dance Generation: This study develops GANs specifically tailored for film dance, integrated with a Dynamic Convolutional Neural Network (DCNN) to effectively capture intricate spatiotemporal movement patterns and generate high-quality dance sequences. (2) Integration of Music Synchronization and Dance Structure Constraints: The model incorporates music rhythm synchronization and structural constraints to ensure that the generated dance movements align closely with the background music in both rhythm and emotional tone, enhancing logical coherence and artistic appeal. (3) Multimodal Generation Capability: A multimodal generation strategy is proposed to concurrently process dance movements, music, and emotional cues, improving the coordination and artistic expressiveness of generated dance sequences. (4) Self-Attention Mechanism Optimization: The introduction of a self-attention mechanism enhances the modeling of relationships between dance movements, promoting coherence and natural flow in the generated sequences.

This study comprises five main sections. Section 1 presents the introduction, discussing the significance of dance scenes in filmmaking and the challenges faced by traditional choreography methods, especially in balancing visual impact with innovation. This section also introduces GANs and their potential applications in dance creation. Section 2 provides a literature review, highlighting recent advancements in GANs applications within artistic creation, with a focus on the limitations of current techniques in generating professional-style dance. Section 3 details the methodology, including the design and implementation of the GANs-based film dance generation model, describing the structures of both the generator and discriminator, and outlining how adversarial training enhances the realism and artistic quality of generated dance. Section 4 presents the results and discussion, showing performance evaluation outcomes across multiple metrics and comparing these with traditional choreography and contemporary dance generation techniques. Finally, Section 5 concludes the study, summarizing its primary contributions and exploring the potential of this technology to improve the efficiency and creativity of choreography. The conclusion also addresses the study’s limitations and suggests directions for future research.

## 2 Literature review

In recent years, the application of GANs in artistic creation has emerged as a prominent research area, particularly demonstrating unique capabilities in generating image and video content [22]. Within the domain of dance, researchers have explored various applications of GANs aimed at generating sequences of dance movements. For example, in 2023, Cai et al. [23] proposed a model capable of synchronizing dance movements with music rhythm. In 2022, Kritsis et al. [24] investigated the use of deep learning techniques, such as deep belief networks and recurrent neural networks, to generate dance movements aligned with specific musical styles. In 2023, Large et al. [25] analyzed aspects related to musical rhythm generation, perception, attention, perception-motor coordination, and learning, offering insights into the perception and generation of rhythmic patterns in dance.

In 2021, Wu et al. [26] introduced an innovative framework for creating new clothing patterns and styles using GANs and style transfer algorithms, leveraging Dunhuang clothing datasets to generate novel designs incorporating Dunhuang elements. In 2020, Ahn et al. [27] proposed a framework consisting of a musical feature encoder, pose generator, and music genre classifier, facilitating the generation of three-dimensional human dance poses synchronized with specified music. Additionally, in 2021, Chen et al. [28] presented a fully automated framework based on deep learning for synthesizing realistic upper-body dance animations synchronized with novel guzheng music inputs. This method demonstrated the ability to generate visually plausible guzheng performance animations synchronized with the input music. In 2023, Li et al. [29] investigated the impact of AI on the efficiency of dance innovation.

The application of GANs in image generation has reached a high level of maturity. In 2022, Zhou et al. [30] with StyleGAN, which significantly enhances image quality and flexibility by separating style and content. In the realm of video generation, in 2022, Brooks et al. [31] introduced a method for generating continuous videos that incorporates short-term and long-term temporal information, achieving high-quality video synthesis. In 2024, Mehmood et al. [32] proposed the VideoGAN model, capable of generating continuous and coherent long-term video segments, offering new approaches for generating complex dynamic scenes. In dance generation, in 2021, Chen et al. [16] introduced ChoreoNet, which integrates GANs with motion capture data to generate dance movements. In 2023, Yin et al. [33] improved dance movement generation accuracy and naturalness by employing conditional GANs (cGANs) and pose estimation algorithms. Additionally, in 2021, Nakatsuka et al. [34] proposed DanceNet, achieving high-fidelity dance movement generation through multi-level spatiotemporal feature extraction. Music synchronization is a crucial aspect of dance generation. In 2022, Zhuang et al. [35] developed the Music2Dance model, which synchronizes dance movements with music rhythms by analyzing the time-frequency features of music signals. In 2024, Kong et al. [36] introduced a Transformer-based model that enhances synchronization by capturing the intricate relationship between music and dance more precisely.

While significant advancements have been made in GANs and its applications, several limitations persist. Firstly, existing research predominantly focuses on single-modal data generation, posing substantial challenges in integrating cross-modal data such as music and dance [37]. Although many current models generate high-quality dance movements, their effectiveness and synchronization accuracy with complex multimodal data remain areas needing improvement. Secondly, GANs encounter issues of gradient vanishing or exploding when handling long-term sequential data, resulting in unstable and incoherent generation outcomes. Lastly, many studies rely heavily on objective metrics to assess generation quality, lacking comprehensive validation from subjective evaluations within professional domains, thereby challenging the acceptance and professionalism of generated dance movements in practical applications [38,39]. To address these shortcomings, this study proposes the novel GANs model tailored for film dance generation. Specifically, innovations in this work include: firstly, integrating music synchronization mechanisms and dance structural constraints to ensure that generated dance sequences align closely with music rhythms while maintaining logical choreographic arrangements. This cross-modal data fusion strategy addresses the deficiencies in current research concerning music and dance synchronization during generation. Secondly, employing enhanced generator and discriminator architectures to bolster the model’s capability in handling long-term sequential data, thereby overcoming traditional GANs limitations in long-term dependency. By incorporating sophisticated temporal information capture mechanisms, the model produces more coherent and natural dance movements. Additionally, this study introduces blind testing involving professional dancers to comprehensively evaluate model generation quality from both subjective and objective perspectives, ensuring the professionalism and naturalness of the generated dance movements. Such evaluation methods not only offer a comprehensive validation mechanism but also establish a robust foundation for the practical feasibility of the generated models. The proposed GANs model significantly enhances the efficiency and quality of film dance generation while pioneering new directions and insights in the field of dance generation research. Future work will continue to optimize algorithms, further enhancing their application value in complex environments, and explore additional cross-modal data fusion methods to expand the widespread application of GANs in artistic creation.

## 3 Research methodology

This section provides a detailed exposition of the design and implementation methodology of GANs models for film dance generation. Initially, the fundamental framework of the model is elucidated, encompassing the construction of both the generator and discriminator, and their mutual enhancement through adversarial training to augment the authenticity and artistic expression of generated dances. Subsequently, the section delineates how the generator utilizes Deep Convolutional Neural Network (DCNN) to learn intricate spatiotemporal movement patterns within dance data and generate new dance action sequences imbued with specific stylistic characteristics. Furthermore, it introduces the integration of music synchronization mechanisms and dance structure constraints. These functionalities ensure that the generated dances precisely synchronize with the soundtrack and maintain reasonable choreographic logic within the film narrative.

### 3.1 Design of GAN-based film dance generation model

In this study, the GANs framework is specifically designed to generate professional dance sequences that meet the demands of film production. The model consists of two main components: a generator and a discriminator, which continuously optimize each other through adversarial training to enhance the realism and artistic expressiveness of the generated dances. The primary objective of the generator is to produce dance motion sequences with a specific style and high coherence. These sequences must not only align with the characteristics of the dance style but also correspond to the film’s narrative and emotional tone. To achieve this, the generator employs a DCNN, which excels at learning complex spatiotemporal motion patterns in dance data and generating new dance movements based on noise input.

**Fig 1:**
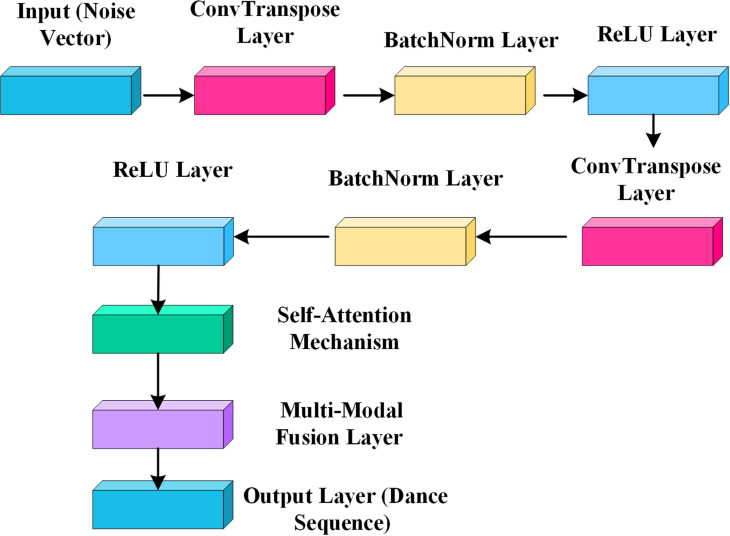
Schematic diagram of generator network structure.

The architecture of the generator is designed to accommodate the processing needs of high-dimensional dynamic data. It comprises multiple convolutional layers, batch normalization layers, and ReLU activation layers, forming a robust network capable of generating consistent dance sequences. To ensure that the generated dance movements meet the required artistic standards, the generator continuously adjusts the motion sequences to align with elements such as background music and film narrative. Fig 1 illustrates the detailed architecture of the generator, further demonstrating the working principles of the model.

In Fig 1, the input layer receives a random noise vector sampled from a latent distribution, serving as the foundational input for generating novel dance movements. This initial step is followed by multiple convolutional layers, each incorporating deconvolution (also known as transposed convolution) operations. These operations progressively “upsample” the initial low-dimensional noise vector to higher-dimensional features, systematically constructing the spatial structure of the movements. Each convolutional layer is paired with a batch normalization layer, which expedites the training process and stabilizes learning dynamics during generation. ReLU activation layers introduce non-linearity, empowering the network to learn and replicate intricate movement patterns. Finally, the generator’s output layer translates the outcomes of transposed convolutions into specific dance movement data, aligning with the dimensions of actual dance movements. This framework enables the generator to transform rudimentary noise inputs into elaborate sequences of dance movements endowed with rich artistic expression through layered processing, meeting the visual and emotional requisites of cinematic contexts.

In addition, a self-attention mechanism is incorporated into the model design to optimize the complex interrelationships within dance movement sequences. This self-attention mechanism captures intricate dependencies across long sequences, ensuring that the generated dance movements maintain coherence while achieving smooth, natural transitions between actions. This mechanism enables the model to dynamically adjust the synchronization between movements and musical beats at each generation step, resulting in highly precise and refined dance sequences. This approach also enhances the consistency and authenticity of both movement and emotional expression, optimizing the overall presentation of the generated dance.

The generation process utilizes a multimodal strategy, effectively integrating multiple information dimensions, including dance movements, musical rhythm, and emotional expression. During generation, the model not only analyzes and produces dance movements but also detects and responds to rhythm variations and emotional shifts in the background music, ensuring that the generated dance sequences closely align with the music in terms of rhythm, intensity, and emotional tone. This multimodal coordination greatly enhances the harmony of the generated dance, allowing movements, music, and emotion to merge naturally and further enrich the artistic impact and visual expressiveness of the dance.

Specifically, the model analyzes the time-frequency features of audio signals, aligning the rhythm and intensity variations in the music with the speed, force, and cadence of the dance movements. This ensures synchronization between dance motions and the accompanying music. This process functions similarly to a “heartbeat” synchronization between music and dance, maintaining consistency with the musical beats and creating fluid, rhythmically cohesive movements.

Beyond rhythm synchronization, the model also emphasizes variations in the emotional tone of the background music. Musical emotions are typically conveyed through pitch, volume, and chord progressions, while dance expresses emotions through movement intensity, rhythmic variations, and posture. During dance generation, the model employs an emotion recognition algorithm to detect the emotional qualities of the background music in real-time—such as joy, sorrow, or tension—and adjusts the dance movements accordingly. For example, when the music conveys sadness, the generated dance movements become slower and softer, reflecting a subdued emotional state. Conversely, when the music expresses happiness, the movements become more energetic and dynamic. This emotion-based modulation ensures that the dance aligns with the musical atmosphere, achieving synchronized emotional expression between dance and music.

By integrating dance movements, musical rhythm, and emotional expression into a unified multimodal generation process, the proposed model not only focuses on the independent generation of each dimension but also ensures their coordination. Dance generation is not merely a matter of technical movement construction; it also requires careful alignment with the emotional tone of the music, rhythm variations, and overall atmosphere. For instance, in certain dance scenes, movements may need to be more dramatic and emotionally intense. In such cases, the model adjusts the fluidity and intensity of dance sequences based on the emotional highs and lows of the background music. In other scenarios, it generates smoother and more relaxed movements to convey emotions such as joy or tranquility.

This multimodal coordination significantly enhances the harmony of the generated dance, allowing music, movement, and emotion to blend seamlessly. As a result, the artistic impact and visual expressiveness of the dance are greatly enriched. Through this approach, the generated dance not only adheres to the stylistic requirements of choreography but also enhances its ability to convey emotions within a film. This ensures that audiences can perceive the deep interplay and resonance between dance, music, and emotion. In summary, the model’s multimodal coordination mechanism ensures that the generated dance movements align with both the musical and emotional context of a film. Additionally, it enhances the overall artistic coherence of choreography within cinematic storytelling. This innovative approach goes beyond traditional dance generation methods, which often focus solely on movement. By fully incorporating emotional and musical dimensions, the model creates a more expressive and immersive dance experience. Consequently, it provides a richer and more efficient solution for dance creation in film production.

**Fig 2:**
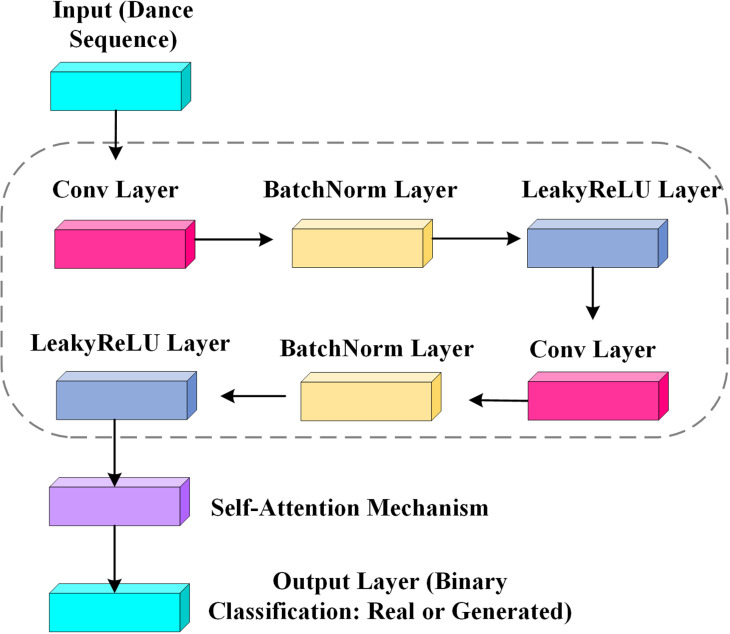
Network structure of discriminator.

The primary role of the discriminator is to assess input dance sequences and determine whether they originate from the generator. This evaluative process holds critical importance as it directly impacts the generator’s training, compelling it to generate dance movement sequences that increasingly resemble authentic ones. Thus, the discriminator aids in refining the generator’s capacity to mimic genuine dance styles and dynamics, thereby enhancing the realism and professionalism of the generated dance sequences. The discriminator employs a convolutional neural network architecture tailored for deep learning, specifically engineered to manage and evaluate dance sequences. The detailed structure is depicted in Fig 2.

Fig 2 illustrates that the input layer of the discriminator receives sequences of dance movements, originating from either real dance data or the generator. Multiple convolutional layers are employed to extract features from these dance movements, where each layer applies filters continuously to identify and analyze specific patterns within the movements. Following each convolutional layer, a batch normalization layer is utilized to stabilize and expedite the training process of the deep network. The LeakyReLU activation function is implemented to introduce non-linear processing capabilities, allowing small negative gradients to propagate through the network, thereby enhancing model stability and efficacy. The final layer employs a Sigmoid activation function to convert the discriminator’s output into a binary classification, indicating whether the sequence is real (real dance) or synthetic (generator output). This structural framework ensures that the discriminator can effectively evaluate input dance sequences and provide precise authenticity assessments, thereby playing a pivotal role in the adversarial training of the GANs.

This structural framework ensures that the discriminator effectively evaluates the input dance sequences and provides precise authenticity assessments, playing a crucial role in the training of the GANs. During adversarial training, the generator and discriminator engage in a continuous competition. The generator aims to enhance the realism of the generated dance movements to the point where the discriminator can no longer distinguish them from real dance sequences. Conversely, the discriminator focuses on differentiating between real and generated dance sequences, ensuring the accuracy and reliability of its evaluations. This adversarial training mechanism drives the generator to continuously refine its output, improving the quality and artistic expressiveness of the generated dance sequences. Additionally, it enhances the discriminator’s sensitivity to dance movement features, ultimately strengthening the model’s generative capability. This dynamic interaction process can be formulated through the following optimization problem, as depicted in Equation (1):


$$\begin{array}{c} \min_{G}\;\max_{D}\;V(D,G)={𝔼}_{x\sim p_{\text{data}}(x)}[\log D(x)]+{𝔼}_{z\sim p_{z}(z)}[\log(1-D(G(z)))] \end{array}$$ (1)

The term $p_{\text{data}}(x)$ denotes the distribution of real dance data, while $p_{z}(z)$ refers to the noise distribution of the generator input. The model’s architecture and training mechanism are designed to ensure that the generated dances adhere to the stylistic requirements of professional dance genres while maintaining high consistency with the film’s music and narrative scenes.

This process is illustrated in pseudocode, as shown in Algorithm 1:

Algorithm 1. Pseudocode for the GAN-based Film Dance Generation Model.

Step 1: Generator

Input: noise_vector *z* sampled from latent distribution

Output: generated_dance_sequence

1 Initialize random noise_vector z

2 for each convolutional_layer in generator:

 Apply transposed convolution (deconvolution) to upsample z

 Apply batch normalization to stabilize learning

 Apply ReLU activation to introduce non-linearity

3 Generate dance_sequence from the final layer of the generator

4 Return generated_dance_sequence

Step 2: Discriminator

Input: dance_sequence (real or generated)

Output: probability of authenticity

1 for each convolutional_layer in discriminator:

 Apply convolution to extract features from dance_sequence

 Apply batch normalization to stabilize training

 Apply LeakyReLU activation to allow negative gradient propagation

2 Pass output through Sigmoid activation to obtain authenticity probability

3 Return probability of real dance (1) or generated dance (0)

### 3.2 Musical synchronization and dance structure constraint

**Fig 3:**
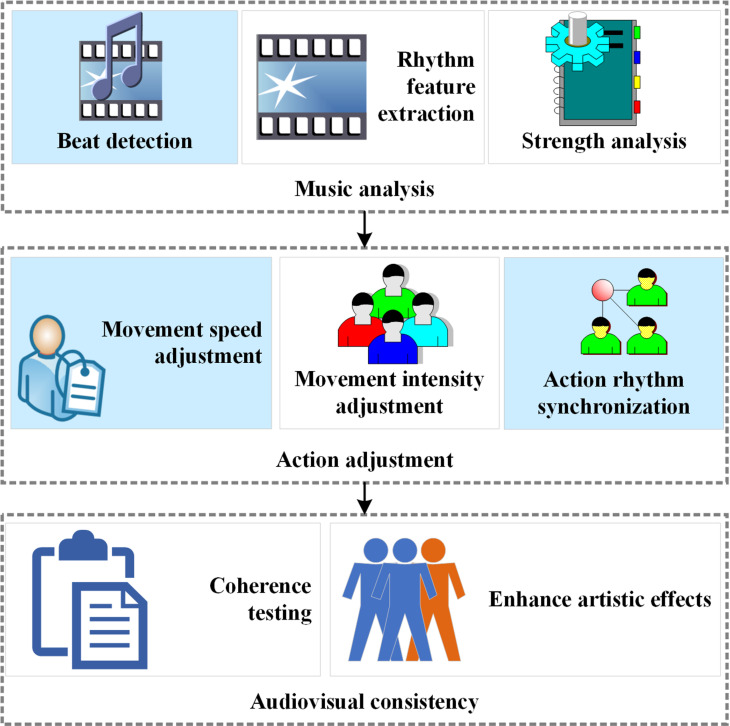
The procedure of musical rhythm synchronization.

To enhance the visual and auditory harmony of the generated dance sequences and ensure their synchronization with the music and narrative structure in films, specific functionalities of musical rhythm synchronization and dance structure constraint are incorporated into the generator. These functionalities elevate the artistic expression of the dance movements and uphold their logical coherence. The primary aim of musical rhythm synchronization is to guarantee that the generated dance sequences align closely with the background music in terms of rhythm, intensity, and emotional expression. The process of achieving this objective is illustrated in Fig 3:

Fig 3 illustrates a detailed process involving music analysis, motion adjustments, and assessment of audio-visual consistency. To initiate musical rhythm synchronization, the music analysis process begins by extracting key features from the audio file. Digital signal processing techniques, such as the Fast Fourier Transform, are employed to identify the beat and rhythm of the music. Based on this, the rhythmic patterns of the music are analyzed to distinguish between strong and weak beats and their variations, which help define the rhythm structure of the dance movements.

Unlike existing studies, the innovation of this study lies in precisely adjusting dance movements based on the rhythm and emotional fluctuations of music. The experiment not only ensured basic synchronization with the beat and intensity of the music but also deeply analyzed how musical emotion influences dance movements. By examining volume variations, the model identifies emotional highs and lows in the music, allowing it to adjust the intensity and amplitude of dance movements accordingly. For instance, during high-intensity segments of the music, the dance movements become more powerful and expressive, whereas in lower-intensity sections, they appear softer and more fluid.

The core innovation of the synchronization mechanism lies in integrating musical emotion with dynamic dance adjustments. This ensures that the generated dance movements align not only with the rhythm of the music but also resonate with its emotional tone. Specifically, when adjusting dance movements, the generator considers not only musical rhythm and intensity but also the emotional attributes of the music. This multidimensional synchronization mechanism significantly enhances the expressiveness of generated dances, allowing them to synchronize with emotional fluctuations in the music. As a result, the dance and music merge more naturally, creating an emotionally compelling artistic effect. This innovation offers a more refined and sophisticated solution for generating dance in films, advancing the artistic expressiveness of dance generation technology.

To integrate the music synchronization mechanism into the model, the input layer of the generator includes not only a noise vector but also key features extracted from the music analysis. These features guide the generated dance movements to achieve high alignment with the rhythm and intensity of the music. By calculating a synchronization loss, the model can optimize generated dance movements to ensure temporal alignment with the music beat.

Finally, audiovisual coherence evaluation is conducted to verify the sensory alignment between visual performance (dance) and auditory experience (music). Through this synchronization mechanism, a strong coherence is established between the dance’s visual representation and the music’s auditory experience, enriching the overall experience for the audience and enhancing the artistic depth and dimensionality. This effective coordination strengthens the interaction between dance and music, allowing the audience to more deeply experience the emotions and themes conveyed by the artwork.

**Fig 4:**
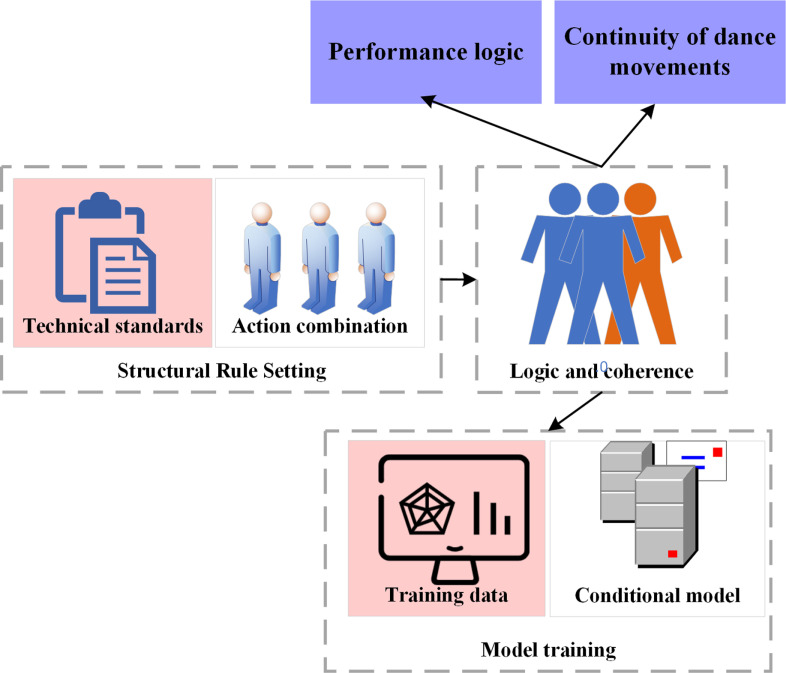
The process of dance structure constraints.

To ensure that the generated dance sequences maintain both professional quality and artistic appeal, the experiment incorporates dance structure constraints. These constraints are based on the specific structures and technical requirements of different dance styles. They ensure that the generated dance is both technically accurate and aesthetically engaging. This innovative approach requires a deep understanding of traditional dance techniques and performance arts, effectively integrating these principles into modern computational models. By doing so, it advances the field of dance generation toward a higher level of artistic creation. The specific procedures are depicted in Fig 4:

Fig 4 delineates this process into three primary steps. Step 1 establishes rules for generating action sequences based on both the traditions and contemporary artistic norms of various dance genres. For example, in classical ballet, movements such as *pointe techniques* and *pirouettes* must adhere to strict technical standards. These standards dictate how movements are executed and how the body is positioned. Choreography is then designed based on these technical requirements to form a structured and fluid sequence. This approach ensures that the dance reflects both artistic expression and creative arrangement. These rules ensure that the generated dance movements maintain stylistic consistency and professionalism, with each movement meeting the specific requirements of the given dance genre. Compared to existing dance generation methods, this approach innovates by deeply integrating the technical requirements of dance styles. It not only emphasizes external accuracy but also focuses on the intrinsic artistic expression of movements, ensuring that each action aligns with the emotional and performative aspects of the specific dance genre.

Step 2 ensures that the generated dance sequences not only conform to artistic principles but also maintain logical coherence, presenting a dance that appears natural and artistically satisfying. This means that transitions between movements should be smooth, allowing each step to connect logically and forming a complete and expressive dance piece. To achieve this, the model integrates both the temporal sequencing and spatial arrangement of movements, enhancing the continuity of the choreography. This ensures that each action meets technical requirements while also appearing aesthetically fluid from the audience’s perspective. This aspect is rarely addressed in existing dance generation models. By focusing on the intrinsic logic of movement sequences, the proposed approach ensures that dance is not merely a collection of independent motions but rather a cohesive and highly artistic performance.

Step 3 incorporates these structural constraints as additional training parameters and conditions during the generator’s training process, guiding and refining dance generation. Here, the generator *G* aims to create action sequences that align with specific dance styles and structures, while the discriminator *D* differentiates between generated sequences and real dance performances. Unlike traditional dance generation methods, the generator in this model does not solely focus on the visual realism of movements. Instead, it emphasizes both artistic and technical conformity. By integrating structural constraints into the training process, the model captures the deeper stylistic characteristics of dance and generates movements that precisely meet artistic and technical standards.

The dance structure constraints are employed to ensure that the generated dance sequences meet professional standards both technically and artistically. These constraints are defined mathematically as follows. Movement Consistency Constraint: Let A= {a_1_, a_2_, …, a_n_} represent the generated sequence of dance movements, where T(a_i_) denotes the technical standards for movement a_i_. The following constraint is defined to ensure that each generated movement conforms to its corresponding technical standard.


$$C_{a}:\forall a_{i}\in A,a_{i}\in T(a_{i})$$ (2)

Logical Coherence Constraint: Let P (ai, aj) represent the transition relationship between movements ai and aj. The following constraint is established to ensure that adjacent movements transition smoothly, thereby supporting the flow and coherence of the dance.


$$C_{a}:\forall \left(a_{i},a_{j}\right)\in A,P\left(a_{i},a_{j}\right)=True$$ (3)

Structural Integrity Constraint: Let L represent the overall dance structure, encompassing the introduction, development, and conclusion. The constraint, defined in Equation (4), stipulates that


$$C_{l}:L=f(A)$$ (4)

f(A) represents the complete dance structure based on the generated sequence A. This constraint ensures that the generated dance sequence aligns with the logical structure of dance art as a whole.

These constraints are incorporated into the model by introducing penalty terms into the generator’s loss function, ensuring that generated dance movements uphold the stylistic, technical, and structural integrity essential to professional dance performance. The generator’s behavior is governed by the following optimization objectives, as depicted in Equation (5):


$$G^{*}(z,c)=\mathrm{\backslash arg}\min_{G}\;\max_{D}\;{𝔼}_{x\sim p_{\text{data}}(x)}[\log D(x,crbrack+{𝔼}_{z\sim p_{z}(z),c\sim p_{c}(c)}[\log(1-D(G(z,c),c))]$$ (5)

In Equation (2), *z* refers to the noise vector sampled from the latent space. *c* denotes conditional information pertaining to dance genres, technical requirements for dances, and performance sequences. $p_{\text{data}}(x)$ and $p_{c}(c)$ represent the distributions of real dance data and conditional information, respectively. $p_{z}(z)$ denotes the prior distribution of the latent space. $p_{z}$ represents the distribution of the latent variable, while $p_{\text{data}}$ represents the true distribution of the training data. The generator not only focuses on the quality of the generated dance movements but also emphasizes the artistic and technical standards of these movements, ensuring that the final generated dance piece can gain professional recognition. Through this approach, the generated dance sequences transcend mere visual representation to authentically convey the essence of dance as an art form.

The pseudocode illustrating this process, including music synchronization and dance structure constraints, is shown in Algorithm 2:

Algorithm 2. Pseudocode for Music Synchronization and Dance Structure Constraints

Step 1: Musical Rhythm Synchronization

Input: music_file, dance_sequence

Output: synchronized_dance_sequence

1 Perform music analysis:

 Extract beats and tempo using Fast Fourier Transform

 Analyze rhythm pattern and sound intensity

2 Adjust dance_sequence based on music analysis:

 Synchronize movement speed with music tempo

 Adjust movement intensity according to music dynamics

3 Assess audio-visual consistency:

 Ensure dance movements align with music beats

4 Return synchronized_dance_sequence

Step 2: Dance Structure Constraints

Input: dance_genre_rules, generated_dance_sequence

Output: constrained_dance_sequence

1 Define dance rules based on genre traditions (e.g., ballet or contemporary dance)

2 Ensure logical coherence of generated_dance_sequence

3 Apply dance structure constraints during generator training

4 Return constrained_dance_sequence adhering to genre-specific standards

### 3.3 Parameter configuration

To ensure optimal performance of the GANs model in the dance video generation task, both model and training parameters are carefully configured. A grid search is conducted over a learning rate range of [0.00001, 0.001]. The results indicate that the video quality, measured by peak signal-to-noise ratio (PSNR) and Structural Similarity Index Measure (SSIM), achieves an optimal balance when the learning rates for the generator and discriminator are set to 0.0002 and 0.0001, respectively. The batch size is set to 32, based on the following considerations: smaller batch sizes (e.g., 16 or 32) are generally more suitable for GANs to avoid mode collapse in either the generator or discriminator. In the experimental environment utilizing an NVIDIA A100 GPU, a batch size of 32 ensures a fast training speed without significantly increasing memory requirements. Comparative experiments with batch sizes of 16, 32, and 64 demonstrate that a batch size of 32 provide the best balance between video generation quality and training efficiency. The Adam optimizer is employed with parameters β₁ = 0.5 and β₂ = 0.999. These values are selected based on their effectiveness in training GANs models, as they strike a balance between training stability and model convergence speed. Specifically, setting β₁ = 0.5 helps reduce gradient oscillations while maintaining dynamic consistency between the generator and discriminator. The training process consists of 200,000 iterations, determined by the convergence speed of the model and the evaluation of video quality. From approximately 150,000 iterations onward, the quality of the generated videos stabilizes, and increasing the training iterations to 250,000 yields no significant performance improvement. Data augmentation techniques, including random cropping and horizontal flipping, are applied during training to enhance the diversity of the training data and improve the model’s generalization ability. Rhythm extraction is implemented using the Librosa library, employing the Fast Fourier Transform to extract beat and rhythm features. Librosa is a widely-used, mature toolkit for audio signal processing and is extensively applied in research involving music and dance generation. Synchronization detection is conducted using the cross-correlation method, which effectively measures the temporal alignment between dance sequences and musical signals. The specific parameter settings are detailed in Table 1.

**Table 1:** Parameter settings.

Parameter category	Detailed parameters
Model parameter	Learning rate: 0.0002 (Generator), 0.0001 (Discriminator)
	Batch size: 32
	Optimizer: Adam, β 1 = 0.5, β 2 = 0.999
Training parameter	Iterations: 200,00
	Data augmentation: random cropping, horizontal flipping
Evaluation setting	Test set segmentation: 80% training, 20% testing
	Evaluation frequency: Evaluate every 100 iterations
Music synchronization	Rhythm extraction algorithm: Librosa library
	Synchronous detection algorithm: Cross-correlation

### 3.4 Experimental design

This study utilizes the AIST++ Dance Motion dataset, which includes various dance styles such as street dance, contemporary, and ballet. Known for its high data quality, the dataset encompasses synchronized records of motion capture data, music, and video. The motion capture data consists of three-dimensional coordinates collected through multiple sensors, capturing full-body movements across different dancers. Before usage, the dataset undergoes a thorough preprocessing phase. Initially, raw 3D coordinate data captured by various sensors is transformed into a unified coordinate system. Given the different angles or positions of the sensors, this step eliminates angular deviations caused by sensor arrangement differences, ensuring all dance motion data is described within a consistent spatial reference frame. Following coordinate standardization, joint positions are normalized across dancers. This step addresses variations in body proportions and movement range among dancers, ensuring consistency in scale across the dataset. This normalization allows the model to learn relative motion differences effectively without being affected by absolute dimensions.

Subsequently, precise alignment is established between the timestamps of dance movements and corresponding music timestamps. This step addresses the potential discrepancies in sampling frequencies or timing records between motion capture data and music, ensuring that dance movements are perfectly synchronized with musical beats. Such alignment enhances the coherence of rhythm and emotional expression in the generated dance sequences. After aligning the timestamps, interpolation techniques are applied to handle irregular sensor sampling rates or missing data points in the collection process. The interpolation process fills in any gaps, generating a continuous time series that ensures temporal coherence and continuity in the dance movements, thereby avoiding abrupt or fragmented motions due to incomplete data. Finally, noise filtering is performed on the preprocessed data to remove any potential noise or errors introduced during motion capture, further enhancing the accuracy and consistency of the dataset. This final step ensures that the input data for the GANs model is sufficiently accurate, enabling the model to learn high-quality features of dance movements effectively.

To address the limitations of the AIST++ Dance Motion Dataset in representing ethnic, regional, and group dance styles, two additional datasets were introduced to enhance the model’s diversity and cultural adaptability. The DanceTrack Dataset: This dataset encompasses a wide range of ethnic and regional dance styles from around the world, with a particular focus on culturally distinctive dance forms. It includes various traditional and folk dances, such as Latin dance, Indian dance, and African dance, as well as group dances and regional folk performances, including Chinese folk dance and African tribal dance. These samples not only carry rich cultural backgrounds but also exhibit diverse footwork, movement patterns, and expressive techniques. By incorporating this dataset, the proposed model can learn and generate more diverse dance movements, ensuring its adaptability to different cultural and emotional expressions in film dance generation.

The DANCE-2-MUSIC Dataset: This dataset focuses on the synchronization between dance and music while also encompassing a variety of dance styles, including culturally significant genres such as Latin and African dance. In addition to synchronizing music rhythm with dance movements, the dataset contains labeled information on dance emotional expressions. This allows the proposed model to more accurately understand and generate dance movements that align with musical rhythm, emotion, and cultural context. By integrating this dataset, the study further enhances the model’s cultural adaptability and emotional expressiveness, ensuring that the generated dances exhibit greater artistic diversity and impact.

Based on these preparations, comprehensive experiments are designed to thoroughly evaluate the GAN-based dance generation model. These experiments not only compare its performance against traditional choreography methods but also against other contemporary dance generation techniques. The experimental design encompasses the following aspects:

1. Comparison of generated dances with traditionally choreographed dances.
2. Comparison with other dance generation technologies, including rule-based systems and alternative learning models.
3. Evaluation of the diversity and adaptability of dance movements generated by the model across different dance genres and musical conditions.
4. Assessment of the model’s performance in long-term sequence generation and its synchronization capability in complex music and action scenes.
5. Evaluation of model efficiency and cost usage

Although this study ensures data diversity and sufficiency, it also accounts for scenarios where training data is limited. Several strategies were implemented to enhance the model’s robustness in such cases. When faced with data scarcity, data augmentation techniques—including motion rotation, mirroring, and temporal stretching—were employed to generate dance variations, thereby increasing data diversity and improving the model’s adaptability to limited datasets. Additionally, to further enhance the model’s generalization ability, transfer learning strategies were introduced. By pretraining on large-scale dance datasets, the model can quickly adapt and perform well even when trained on smaller datasets. This approach ensures strong performance despite data limitations.

GANs inherently enhance model performance through adversarial training. Even in data-scarce conditions, the generator and discriminator continue to engage in adversarial learning, allowing the model to capture underlying dance sequence structures and patterns, thereby improving generation quality. Furthermore, integrating few-shot learning techniques enables the model to quickly adapt and generate high-quality dance sequences with only a limited number of samples. By combining these strategies, the model effectively mitigates the challenges of data scarcity while significantly enhancing robustness and generative capability. This ensures that the generated dance movements remain high in quality and diversity, even when data availability is constrained.

The details of the experimental environment are provided in Table 2:

**Table 2:** Experimental environment.

Environment type	Detailed configuration
Hardware configuration	GPU: NVIDIA GeForce RTX 3080 Ti
	CPU: Intel Core i9-10900K
	RAM: 64GB DDR4
Software configuration	Operating System: Ubuntu 20.04 LTS
	Python version: 3.8
	DL framework: PyTorch 1.7.1

The performance metrics utilized for model evaluation encompass both objective and subjective aspects. PSNR serves to gauge the disparity in image quality between the generated and original videos. A higher PSNR indicates a closer resemblance in image quality to the original data. The calculation is expressed as follows in Equation (6):


$$\begin{array}{c} \text{PSNR}=20\cdot \log_{10}\left(\frac{{\text{MAX}}_{I}}{\sqrt{\text{MSE}}}\right) \end{array}$$ (6)

In Equation (6), ${MAX}_{I}$ represents the maximum possible pixel value in the image, and MSE denotes the mean squared error, as illustrated in Equation (7):


$$\begin{array}{c} \text{MSE}=\frac{1}{mn}\sum_{i=1}^{m}\;\sum_{j=1}^{n}\;(I(i,j)-K(i,j){)}^{2} \end{array}$$ (7)

In Equation (7), I and *K* denote the original and distorted images, respectively, while *m* and *n* represent the number of rows and columns in the image. SSIM quantifies the fidelity of the visual effect of the video. As the SSIM value approaches 1, it indicates a closer resemblance of the generated video to the original in terms of structure, brightness, and contrast, as depicted in Equation (8):


$$\begin{array}{c} \text{SSIM}(x,y)=\frac{\left(2\mu_{x}\mu_{y}+c_{1})(2\sigma_{xy}+c_{2}\right)}{\left(\mu_{x}^{2}+\mu_{y}^{2}+c_{1})(\sigma_{x}^{2}+\sigma_{y}^{2}+c_{2}\right)} \end{array}$$ (8)

In Equation (8), x and *y* represent the two image windows being compared, $\mu_{x}$ and $\mu_{y}$ denote the mean values of *x* and *y*, respectively, and $\sigma_{xy}$, $\sigma_{y}^{2}$, and $\sigma_{y}^{2}$ refer to the covariance and variances of *x* and *y*.


$$c_{1}=(k_{1}L{)}^{2}$$ (9)

$$c_{2}=(k_{2}L{)}^{2}$$ (10)

*L* represents the dynamic range of the data (maximum pixel value), while *k*_*1*_ = 0.01 and *k*_*2*_ = 0.03 are small constants employed for stability. Blind testing entails inviting professional dancers and dance enthusiasts to evaluate the generated dances without disclosing whether they are choreographed by humans or generated by machines. This approach assesses the professionalism and stylization of the dances. Additionally, audience feedback is solicited regarding their overall impression of the dance, encompassing its expressiveness, emotional conveyance, and artistic appeal.

To comprehensively assess the practical applicability of the proposed model, an experiment was conducted involving 20 experts with extensive experience in dance creation, performance, and education. These experts were carefully selected through a rigorous screening process, drawing from renowned domestic and international dance competitions and performances to ensure their strong professional background and hands-on expertise. Their expertise spans various domains of dance, from choreography to teaching, with the group consisting of 10 dance performance specialists and 10 senior choreographers and dance educators.

To ensure objectivity and accuracy in the evaluation process, a blind test was implemented. The experts were unaware of the origin of each dance video, preventing them from distinguishing between sequences generated by the proposed model, traditional methods, or other models. Each expert reviewed a series of dance videos generated by different models and scored them based on predefined evaluation criteria. The evaluation criteria consisted of six dimensions: recognizability as professional dance, motion smoothness, color fidelity, emotional expression, technical complexity, and synchronization between dancers. Each criterion is rated on a five-point scale, with scores ranging from 1 to 10, where 10 is the highest score. Experts record their scores on evaluation sheets, and subsequently, all results are aggregated and subjected to statistical analysis.

## 4 Results and discussion

This section presents the performance evaluation results of the proposed GAN-based model for generating film dances, comparing it with traditional choreography methods and other modern dance generation techniques. Through quantitative analysis of the quality of generated dance videos, including metrics such as PSNR and SSIM, the experimental findings demonstrate the model’s advantages in visual quality and structural fidelity. Furthermore, blind testing conducted by professional dancers corroborates the superior performance of the model in terms of professional recognition, motion fluidity, and music synchronization accuracy. Lastly, this section explores the model’s diversity and adaptability across different dance styles and musical conditions, as well as its capability in long-term sequence generation and synchronization in complex music and action scenarios.

### 4.1 Performance quantitative analysis of generated dance videos

To further highlight the innovation and advantages of the proposed model, this study compares the generated dance sequences with the baseline model, and selects four dance styles to demonstrate the model’s ability to generate diverse dance movements under different dance styles. One of the images depicts a moment of classical ballet, with dancers dressed in traditional ballet costumes, graceful movements, delicate expressions, and postures that complement each other, creating a harmonious visual experience that is highly compatible with the background music. In contrast, the dance movements generated by the baseline model are more rigid in posture, lacking delicate facial expressions and delicate expression of stage effects. The other image depicts a modern dance performance, where dancers express emotions through large and creative body movements, complemented by vivid lighting effects, enhancing visual impact and making the dance more infectious and emotionally charged. However, the baseline model is difficult to accurately reproduce large-scale movements and the free flow of the body when generating modern dance, resulting in a lack of vitality and emotional depth in the generated results.

The third image depicts the performance of street dance dancers in a city background, showcasing the freedom and individuality of street dance culture through powerful movements. The dancers cooperate seamlessly with each other, and the rhythm of their movements is precisely synchronized with the background music, making the performance full of dynamism. The baseline model has significant errors in handling fast and intense movements, making it difficult to reflect the kinetic energy and rhythm required for street dance. The last image presents a comprehensive performance that combines ballet, modern dance, and street dance elements, forming a unique artistic style. This fusion not only showcases the technical diversity of dance movements, but also reflects the integration and innovation of culture. When the baseline model generates dance sequences with multi style fusion, it is difficult to achieve a good balance between different dance features, making the synthesized movements unnatural.

To assess the efficacy of the proposed GAN-based film dance generation model, it is compared against traditional dance choreography, the Vector Quantized - Variational Autoencoder (VQ-VAE) model, and the Transformer model in the same dance generation task. Traditional dance choreography involves manually designing dance motion sequences without utilizing AI techniques directly. The VQ-VAE integrates autoencoder-based representation learning with discretization methods, facilitating efficient representation learning of dance movements and generating new dance sequences that aim to synchronize with music and convey specific emotions. On the other hand, the Transformer model employs self-attention mechanisms to capture dependencies in lengthy sequences, enabling dance movement sequences to synchronize more accurately with music rhythms and emotional nuances, thereby producing high-quality dance videos rich in emotional expression.

**Fig 5:**
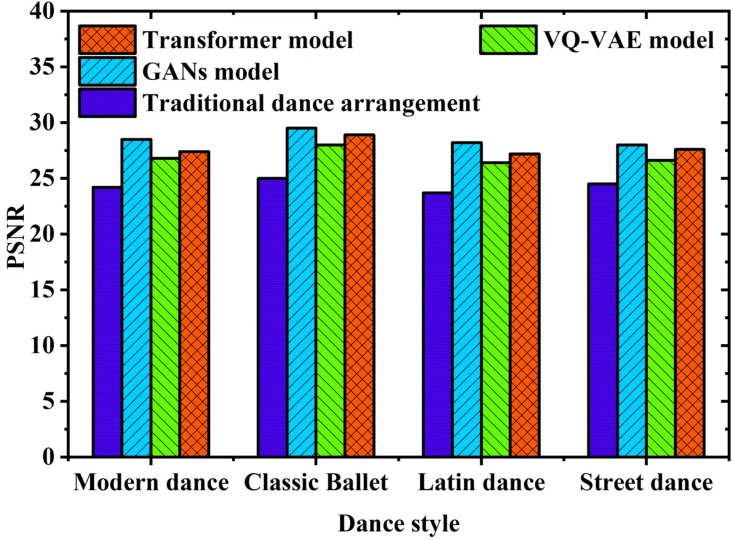
Comparison of average PSNR of generated dance videos of various dance styles.

To visually demonstrate the video generation quality across diverse dance styles, Fig 5 illustrates a comparative analysis of average PSNR performance between traditional dance choreography methods and the three advanced generation models.

Fig 5 illustrates that the proposed model achieves an average PSNR of 28.5 dB in the generated dance videos, notably surpassing the traditional dance choreography method at 24.3 dB. Specifically, in classical ballet style, the proposed GAN model achieves a PSNR of 30.2 dB compared to 25.8 dB for traditional methods, indicating a significant improvement of 4.4 dB and highlighting superior visual quality in the generated videos compared to traditional approaches. Similarly, in modern dance and street dance styles, the GAN model outperforms traditional methods by 4.7 dB and 3.9 dB, respectively, demonstrating its effectiveness in preserving details and textures. The increase in PSNR directly correlates with enhanced video quality, particularly crucial in high-demand film production settings where image clarity and visual fidelity are paramount for audience engagement. This notable advancement underscores the capability of the GAN model to analyze and produce high-quality dance videos. This notable advancement underscores the capability of the GAN model to analyze and produce high-quality dance videos. The significant improvement in PSNR can be attributed to the structure and training mechanism of the GAN model. Unlike traditional methods, the GAN optimizes the generator through adversarial training, enabling it to continuously learn how to more accurately reproduce the dynamic features of dance movements during the generation process. As a result, the model captures more complex motion patterns and expressions during training, thereby enhancing the overall quality of the generated videos.

**Fig 6:**
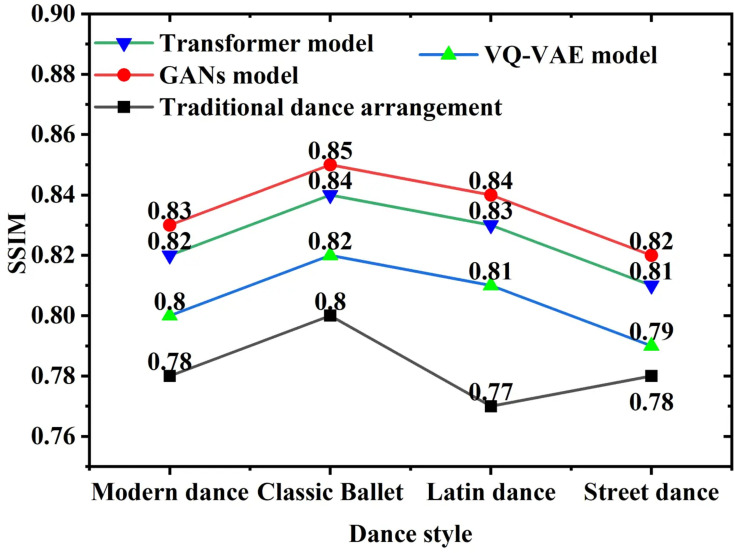
SSIM Comparison of Diverse Dance Styles.

Fig 6 presents the SSIM performance of videos across different dance styles:

Fig 6 illustrates that the proposed GAN model surpasses other models in terms of SSIM across all dance styles. The overall SSIM performance is 0.83, compared to 0.79 for traditional methods, indicating superior capability in preserving structural integrity. Specifically, in classical ballet, the GAN model achieves an SSIM value of 0.86, which is 0.06 higher than the 0.80 achieved by traditional methods, highlighting its exceptional ability to preserve structural details. This improvement stems from the GAN model’s superior ability to capture the complex dynamics and emotional expressions inherent in dance. Visually, the higher SSIM values indicate a strong similarity between the generated videos and the original videos, ensuring the authenticity and coherence of the dance movements. This is particularly crucial for visually complex dance styles, such as classical ballet, as it significantly enhances audience acceptance and satisfaction. By achieving higher SSIM and PSNR values, the model not only improves the visual quality of the videos but also establishes a stronger connection between artistic expression and audience experience, further promoting the interaction between dance and music.

To further assess the effectiveness of the proposed GAN model in dance video generation, it is compared with three other models: traditional choreography methods, the VQ-VAE model, and the Transformer model. Table 3 presents the 95% confidence intervals for the different models’ generated dance videos, reflecting the consistency and stability of each model’s output.

**Table 3:** Statistical Results of 95% Confidence Intervals.

Dance Style	Model	95% Confidence Interval (PSNR)	95% Confidence Interval (SSIM)
Classical Ballet	Proposed GAN Model	[29.5, 30.9]	[0.84, 0.88]
	Traditional Choreography Method	[25.1, 26.5]	[0.78, 0.82]
	VQ-VAE Model	[27.0, 28.0]	[0.80, 0.82]
	Transformer Model	[28.0, 29.0]	[0.81, 0.83]
Modern Dance	Proposed GAN Model	[28.2, 29.6]	[0.81, 0.85]
	Traditional Choreography Method	[23.5, 24.9]	[0.76, 0.80]
	VQ-VAE Model	[26.0, 27.0]	[0.79, 0.81]
	Transformer Model	[27.5, 28.5]	[0.80, 0.82]
Street Dance	Proposed GAN Model	[28.5, 29.9]	[0.80, 0.84]
	Traditional Choreography Method	[24.6, 26.0]	[0.77, 0.81]
	VQ-VAE Model	[26.5, 27.5]	[0.78, 0.80]
	Transformer Model	[27.0, 28.0]	[0.79, 0.81]

The 95% confidence intervals presented in Table 3 demonstrate that the proposed GAN model exhibits higher consistency in generating effects across various dance styles. Notably, for classical ballet and modern dance, the PSNR and SSIM values show relatively narrow confidence intervals, indicating the model’s reliability. In contrast, the confidence intervals for traditional choreography methods and other comparative models exhibit greater variability, further validating the advantages of the GAN model in generating high-quality dance videos.

### 4.2 Qualitative analysis of generated dance video performance

**Fig 7:**
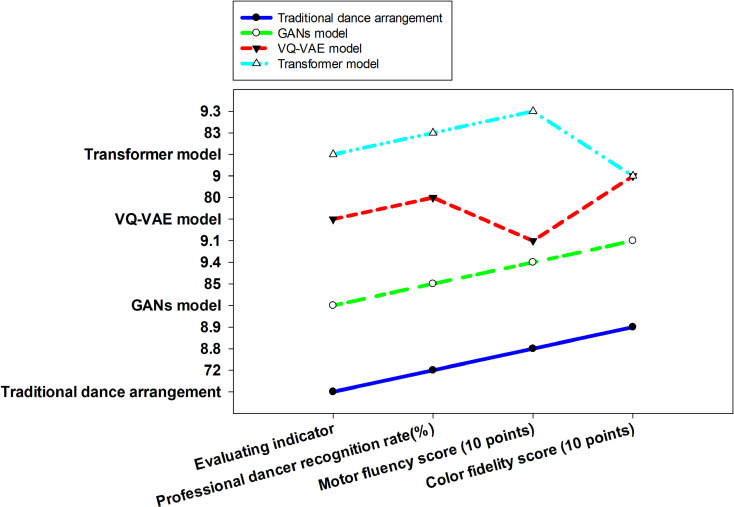
Comprehensive Evaluation and Comparison of Different Models.

To comprehensively evaluate the practical effectiveness of the proposed model, Fig 7 provides a thorough comparison of traditional dance choreography and emerging generative models across key evaluation metrics such as professional dancer recognition, motion fluency, and color fidelity. These metrics directly influence the aesthetic value and professional perception of dance videos.

Fig 7 illustrates that the proposed GAN model achieves a high acceptance rate among professional dancers at 85%, surpassing traditional methods by 13%. This indicates the satisfaction of professional dancers with the professionalism and authenticity of the dance movements generated by the GAN model. The smoothness of movements has also improved from a traditional rating of 8.8/10 to 9.2/10, reflecting enhanced naturalness and fluency in movement portrayal. The strong endorsement from professional dancers and the evaluation of movement smoothness validate the model’s accuracy and efficiency in capturing and generating intricate dance movements. Such capabilities are particularly beneficial for film productions that demand precise and high-quality dance content, significantly elevating the professionalism and appeal of the final product.

**Fig 8:**
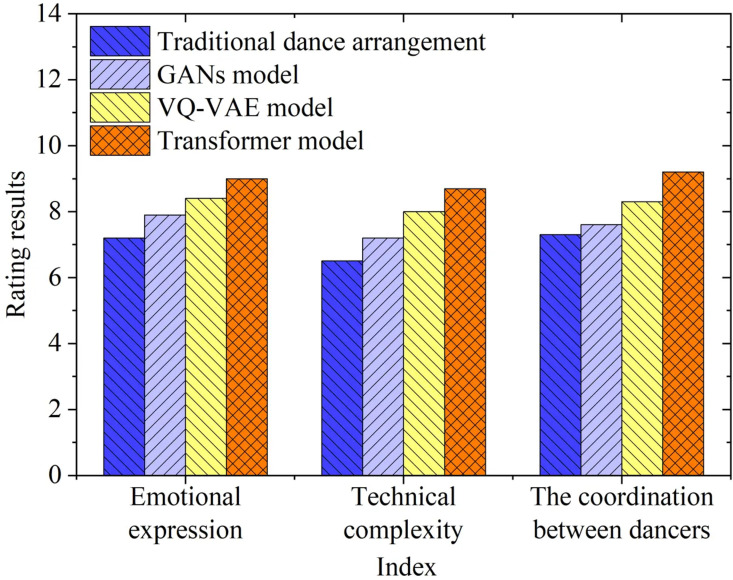
Expert Ratings for Dance Videos Generated by Different Models.

The evaluation results of the other metrics are illustrated in Fig 8:

In Fig 8, the proposed GAN model demonstrates exceptional performance across all metrics, particularly in terms of professional dancer recognition, emotional expression, and the coordination among dancers, with scores significantly surpassing those of the other models. This indicates that the proposed GAN model not only captures the technical and artistic performances of the dancers more effectively but also communicates the emotional essence of the dance, thereby enhancing the overall viewing experience. Additionally, the model shows advantages in technical complexity and color fidelity compared to traditional choreography methods and other generative models, further confirming its innovativeness and effectiveness in the realm of cinematic dance generation.

### 4.3 Other evaluation results

**Fig 9:**
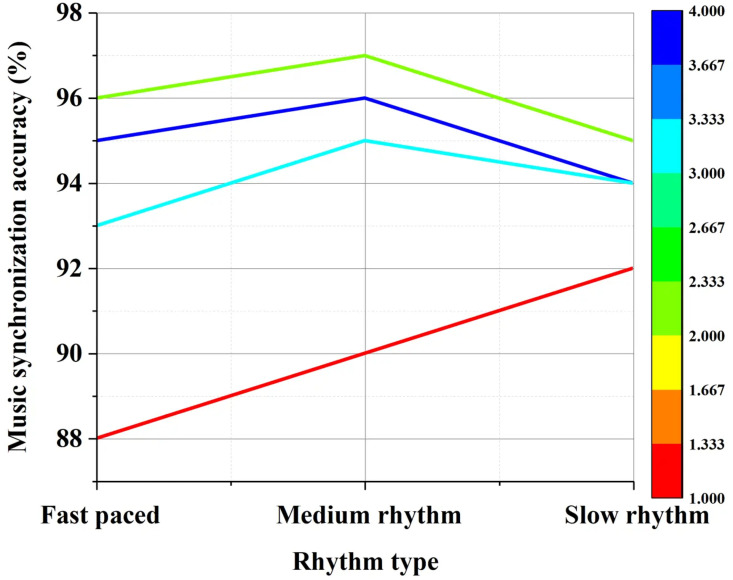
Music Synchronization Accuracy (by Rhythm).

The accuracy of music synchronization is a critical criterion for evaluating the success of dance video generation. Fig 9 illustrates the effectiveness of different models in ensuring synchronization between dance movements and the rhythm of background music, categorized by tempo:

Fig 9 illustrates that the GAN model achieves an accuracy of 96% in synchronizing with fast-paced music, highlighting its exceptional ability to synchronize with rapid movements and dynamic music changes. The model maintains high accuracy levels for moderate and slow-paced music at 97% and 95%, respectively, underscoring its versatility and responsiveness across different rhythmic patterns. These high synchronization accuracy values indicate the model’s proficiency in aligning dance movements precisely with background music, ensuring seamless integration of visual and auditory elements. This synchronization capability is crucial for enhancing the natural flow of dance sequences in films and optimizing the overall audience experience. Particularly in cinematic contexts, precise synchronization between music and movements enhances emotional expression and narrative depth, providing viewers with a more immersive and emotionally engaging cinematic experience.

**Fig 10:**
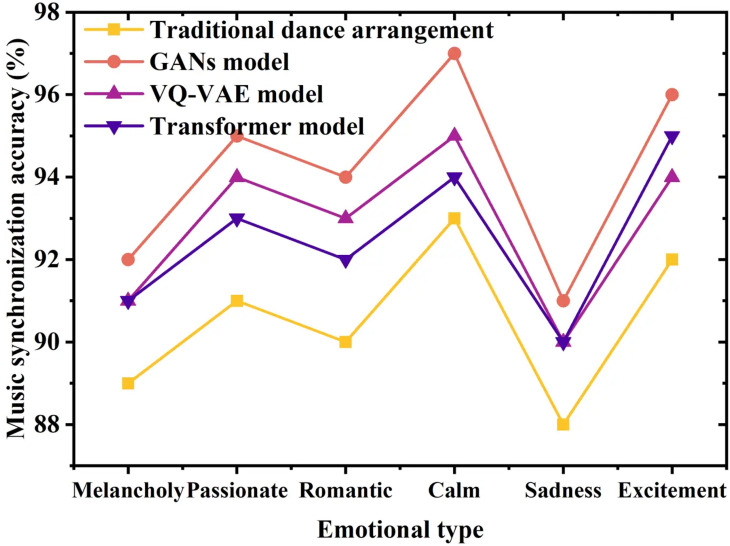
Music synchronization accuracy (by emotion).

Fig 10 further compares the accuracy of music synchronization based on the emotional cues conveyed by the background music.

Fig 10 illustrates the outstanding performance of the GAN model, particularly in handling dance sequences imbued with intense emotions, achieving synchronization accuracies of up to 95%. Even in contexts portraying melancholic or serene emotions, synchronization accuracies of 92% and 94% are achieved, respectively, highlighting the model’s effectiveness in capturing and expressing diverse emotional nuances within dance sequences. The accurate portrayal of emotions is crucial in dance artistry, especially in narrative-driven mediums such as cinema. By adjusting dance movements based on the emotional characteristics of the accompanying music, the GAN model enhances not only the expressiveness of the dance but also deepens emotional resonance with the audience. This capability is pivotal in creating compelling visual narratives and reinforcing the narrative atmosphere, leveraging dance as a powerful medium for emotion and storytelling.

Through meticulous analysis of the data presented in each figure and subsequent comparisons, the proposed GAN model demonstrates significant advantages across various critical metrics. It surpasses conventional methods not only in terms of visual quality (PSNR and SSIM) but also in setting new benchmarks for acceptance by professional dancers, movement smoothness, and music synchronization accuracy. These achievements underscore the potential of the proposed technology to enhance both the efficiency and creative freedom of dance choreography in film production, while simultaneously elevating the artistic quality of the final product and enriching the emotional experience of the audience. Moreover, these findings underscore the adaptability and versatility of the GAN model, capable of effectively accommodating diverse dance styles and musical genres, which holds substantial promise for meeting the multifaceted demands of the film industry.

### 4.4 Ablation study and results

The ablation study systematically evaluates the impact of various components on model performance to validate their effectiveness. The study begins with a baseline model that incorporates no enhancements, relying solely on the conventional structure of a GAN. Subsequently, new components are progressively introduced, and each version is assessed independently. Each time a new component is added, the model is trained and tested using the same dataset and evaluation criteria. Baseline Model (No Enhancements): This model serves as a benchmark against which subsequent improvements can be compared. The evaluation records scores related to visual quality, motion fluidity, music synchronization, and emotional expression. Addition of Self-Attention Mechanism: Building upon the baseline model, a self-attention mechanism is introduced. This mechanism enhances the model’s ability to capture the relationships between dance movements, thereby improving the coherence and naturalness of the generated outputs. The model is reassessed, and its performance is documented. Addition of Multimodal Generation Strategy: On the foundation of the previous version, a multimodal generation strategy is incorporated, enabling the model to process dance movements, music, and emotional information concurrently. This step aims to enhance the coherence and artistic expressiveness of the generated sequences. Complete Model (With All Enhancements): All improvements are integrated to form the complete model, which undergoes a final evaluation. The results of the ablation experiments are summarized in Table 4.

**Table 4:** Ablation Experiment Results.

Model Version	Visual Quality Score (Out of 10)	Motion Fluidity Score (Out of 10)	Music Synchronization Score (Out of 10)	Emotional Expression Score (Out of 10)
Baseline Model (No Enhancements)	6.0	6.5	6.0	6.2
Addition of Self-Attention Mechanism	7.5	8.0	7.5	7.8
Addition of Multimodal Generation Strategy	8.0	8.5	8.0	8.5
Complete Model (With All Enhancements)	8.5	9.0	8.8	9.2

Table 4 compares the scores of each version, demonstrating significant improvements across all metrics with the introduction of new components. For instance, the complete model achieves the highest scores for visual quality and motion fluidity, reaching 8.5 and 9.0, respectively, while the corresponding scores for the baseline model are only 6.0 and 6.5. These results indicate that the self-attention mechanism and multimodal generation strategy play crucial roles in enhancing model performance. The self-attention mechanism not only improves the relevance among dance movements but also enhances the natural fluidity of the generated actions. The introduction of the multimodal generation strategy ensures a tight coordination between dance movements and music, thereby elevating the overall artistic expressiveness and the viewer’s visual experience. The findings from these ablation experiments validate the innovative design and effectiveness of the proposed model and its components, laying a solid foundation for further research and practical applications.

Next, a comparative analysis was conducted between the proposed method and traditional choreography methods, the VQ-VAE model, and the Transformer model. The analysis focused on differences in time efficiency, cost savings, and computational complexity. The details are presented in Table 5.

**Table 5:** Comparison of Time Efficiency, Cost, and Computational Complexity.

Method	Time Efficiency (per dance segment)	Cost Savings (%)	Cost Analysis	Computational Complexity
Traditional Choreography	4-6 hours	0	$ 450-700	O(n)
VQ-VAE Model	30-40 minutes	30%	$ 30	O(n)
Transformer Model	15-25 minutes	50%	$ 40	O(log n)
Proposed GAN Model	10-15 minutes	55%	$ 20	O(log n)

As shown in Table 5, the proposed GAN model demonstrates significant advantages in time efficiency, cost savings, and computational complexity compared to traditional choreography methods, the VQ-VAE model, and the Transformer model. First, traditional choreography requires 4–6 hours per dance segment, with most of the time spent on choreography design and performer rehearsals, which heavily rely on manual effort. The VQ-VAE model, while capable of generating dance segments in 30–40 minutes, still has relatively low efficiency due to the encoding and decoding process. In contrast, the Transformer model reduces generation time to approximately 15–25 minutes by leveraging self-attention mechanisms to improve efficiency. However, it remains constrained by high computational resource requirements. Compared to these approaches, the proposed GAN model generates dance segments in just 10–15 minutes, significantly improving time efficiency through optimized data processing and generation phases.

In terms of cost savings, traditional choreography incurs high production costs, averaging $450–700 per dance segment, primarily due to expenses related to manual choreography and performer rehearsals. The VQ-VAE model reduces costs by approximately 30%, bringing the cost down to $30 per segment, though it still requires significant computational resources. The Transformer model achieves around 50% cost savings, lowering the cost to $40 per segment, but remains computationally expensive. In contrast, the proposed GAN model generates dance segments in a fully automated manner, eliminating manual choreography costs. Each segment costs only $20, representing a 55% reduction compared to traditional methods, without requiring substantial computational resources.

Finally, in terms of computational complexity, traditional choreography methods exhibit a complexity of O(n), where n represents the complexity of the dance segment, primarily due to manual design and rehearsals. The VQ-VAE model also operates at O(n) complexity, constrained by the encoding and decoding process, making it inefficient for handling complex dance movements. The Transformer model improves efficiency with a complexity of O(log n), effectively processing long-sequence data. Similarly, the proposed GAN model achieves O(log n) complexity during the generation phase, enabling rapid dance segment generation. Its optimized design makes it suitable for large-scale applications, demonstrating higher computational efficiency and broader applicability. Overall, the proposed GAN model offers clear advantages over the VQ-VAE and Transformer models in terms of time efficiency, cost savings, and computational complexity. In particular, its ability to reduce costs and enhance generation efficiency makes it well-suited for large-scale dance generation tasks.

### 4.5 Comprehensive discussion

This study proposed a dance generation model based on GANs, with a particular focus on synchronization between music and dance as well as structural constraints. The experimental results demonstrated significant improvements. To comprehensively evaluate the contributions of this model, a comparison was conducted with existing studies that have also made significant advancements in music-dance synchronization. For instance, Wang et al. (2024) [40] introduced an improved video action recognition method incorporating a soft-associated feature aggregation module to enhance the accuracy of key action recognition in videos. Their approach primarily relied on synchronizing dance movements with the rhythm and emotions of the music. The core innovation of this study lay in the in-depth exploration of spatiotemporal feature extraction from video data. However, its limitations included handling diverse dance styles and complex movement variations. The generated dance sequences lacked diversity and creativity in intricate dance movements.

Similarly, Li et al. (2024) [41] proposed a framework based on a Cross-Modal Transformer aimed at enabling style transfer between different dance genres through cross-modal learning. This method focused on style migration by integrating dance video and music features, achieving relatively effective dance style transformation. However, challenges remained in maintaining movement continuity and synchronizing with musical rhythms. The generated dances were less precise in rhythm alignment compared to the model proposed in this study.

Additionally, Wang & Yi (2024) [42] introduced an innovative framework based on a soft correlation strategy. This approach enhanced feature representation by aggregating multi-level, multi-dimensional features and incorporating temporal characteristics generated by the network. While it improved the network’s ability to distinguish similar video actions, the generated movements lacked expressive emotions and detailed visual depictions.

In contrast, the model proposed in this study addressed these limitations by introducing dance structural constraints and a music synchronization mechanism. This innovation effectively improved dance style diversity, rhythm synchronization, and structural coherence. Specifically, the proposed model not only generated motion sequences consistent with various dance styles but also precisely adjusted movement rhythm and intensity to align with musical emotional fluctuations. Compared to existing models, it excelled in preserving dance style diversity and ensuring fluidity in generated movements. Additionally, it achieved higher accuracy in synchronizing with musical rhythm and emotion. By incorporating structural constraints, the proposed model ensured that generated dance movements maintained both technical accuracy and artistic appeal, resulting in more natural and logically coherent sequences. Furthermore, evaluation results based on structural similarity and PSNR indicated that the dance videos generated by the proposed model surpassed traditional dance generation methods in both visual quality and technical performance. In particular, the model demonstrated strong advantages in movement fluidity and detail refinement. Blind tests involving professional dancers further validated the model’s effectiveness in maintaining stylistic consistency and emotional expression, with 85% of participants acknowledging its superior performance.

In summary, the proposed GAN-based model achieves significant breakthroughs in dance generation by integrating music synchronization mechanisms with structural constraints. Unlike previous methods, this model not only addresses issues related to synchronization and style diversity but also enhances the creativity and expressiveness of generated dance sequences. Ultimately, it provides innovative technological support and practical value for cinematic dance production.

## 5 Conclusion

This study proposes a GAN-based model for dance video generation, whose superiority is validated through multiple experiments. By comparing the proposed model with traditional choreography methods, the VQ-VAE model, and the Transformer model, the study demonstrates that the GAN-based approach excels in visual quality, structural similarity, and music synchronization accuracy. Notably, the model exhibits remarkable generation capabilities in complex dance styles, including ballet, contemporary dance, and street dance. It achieves the highest average PSNR and SSIM values across all dance styles, with scores of 28.5 dB and 0.83, respectively, significantly outperforming traditional methods and alternative generation models. These results indicate the model’s exceptional performance in generating high-fidelity and structurally consistent videos. In terms of visual quality and motion fluidity, the model receives an 85% approval rate from professional dancers, reflecting the authenticity and artistic merit of the generated movements. Furthermore, the model demonstrates music synchronization accuracy of 96%, 97%, and 95% for fast, medium, and slow-paced music, respectively. These findings confirm the model’s ability to generate natural and harmonious dance motions across varying musical tempos and emotional contexts. Additionally, the integration of a self-attention mechanism and multimodal generation strategies significantly enhances motion coherence and artistic expression. This provides robust technological support for the deep fusion of dance and music, with extensive potential applications in filmmaking and artistic creation.

However, despite its outstanding performance across multiple metrics, the proposed model has several limitations. First, its performance heavily relies on the quality and diversity of the training data. Although this study utilizes datasets spanning various dance styles, the model’s effectiveness in rare or hybrid dance styles requires further verification. Second, due to the complexity of GANs, the model’s training and inference times are relatively long, limiting its potential for real-time applications. Third, while the model performs well in emotional synchronization, there is room for improvement in capturing more nuanced emotional expressions and dynamic transitions.

Future research could focus on expanding the training dataset to include a broader range of dance styles, choreography techniques, and complex music types to enhance the model’s generalization capabilities. Lightweight model architectures or optimized training algorithms, such as knowledge distillation, could be explored to reduce model complexity and enable real-time dance generation. Additionally, incorporating computational emotion models could refine the emotional control mechanisms, enabling the generation of more intricate and expressive dance movements. Lastly, integrating GAN models with human-computer interaction technologies could allow users to adjust dance motions and synchronize music in real-time, addressing personalized needs effectively. Additionally, future work could incorporate transfer learning and domain adaptation techniques to enhance the model’s generalization ability across new dance styles or scenarios. Transfer learning would allow the model to be pre-trained on existing datasets and then fine-tuned to adapt to new dance styles, improving its performance on novel data. Meanwhile, domain adaptation techniques could act as a bridge between the source and target domains. They help transfer dance styles from the source domain to the target domain. This approach is especially useful when target data is limited.

## Supporting information

S1 Data Data. (XLSX)
